# Breast cancer mortality and overdiagnosis after implementation of population-based screening in Denmark

**DOI:** 10.1007/s10549-020-05896-9

**Published:** 2020-08-30

**Authors:** Elsebeth Lynge, Anna-Belle Beau, My von Euler-Chelpin, George Napolitano, Sisse Njor, Anne Helene Olsen, Walter Schwartz, Ilse Vejborg

**Affiliations:** 1 Nykøbing Falster Hospital, University of Copenhagen, Ejegodvej 63, 4800 Nykøbing Falster, Denmark; 2 Pharmacologie Médicale, Faculté de Médecine, Université Paul-Sabatier III, CHU Toulouse, UMR INSERM 1027, 37 allées Jules Guesde, 31000 Toulouse, France; 3 Department of Public Health, University of Copenhagen, Øster Farimagsgade 5, 1041 Copenhagen K, Denmark; 4 Department of Public Health Programmes, Randers Regional Hospital, Randers, Denmark; 5 Department of Clinical Medicine, Aarhus University, Aarhus, Denmark; 6 Present Address: Novo Nordisk A/S, Bagsværd, Denmark; 7 Breast Cancer Screening, Odense University Hospital, Odense, Denmark; 8 Radiology Department, Copenhagen University Hospital Rigshospitalet, Copenhagen, Denmark

**Keywords:** Breast cancer, Screening, Mortality, Incidence

## Abstract

**Introduction:**

Service breast cancer screening is difficult to evaluate because there is no unscreened control group. Due to a natural experiment, where 20% of women were offered screening in two regions up to 17 years before other women, Denmark is in a unique position. We utilized this opportunity to assess outcome of service screening.

**Materials and methods:**

Screening was offered in Copenhagen from 1991 and Funen from 1993 to women aged 50–69 years. We used difference-in-differences methodology with a study group offered screening; a historical control group; a regional control group; and a regional–historical control group, comparing breast cancer mortality and incidence, including ductal carcinoma in situ, between study and historical control group adjusted for changes in other regions, and calculating ratios of rate ratios (RRR) with 95% confidence intervals (CI). Data came from Central Population Register; mammography screening databases; Cause of Death Register; and Danish Cancer Register.

**Results:**

For breast cancer mortality, the study group accumulated 1,551,465 person-years and 911 deaths. Long-term breast cancer mortality in Copenhagen was 20% below expected in absence of screening; RRR 0.80 (95% CI 0.71–0.90), and in Funen 22% below; RRR 0.78 (95% CI 0.68–0.89). Combined, cumulative breast cancer incidence in women followed 8+ years post-screening was 2.3% above expected in absence of screening; RRR 1.023 (95% CI 0.97–1.08).

**Discussion:**

Benefit-to-harm ratio of the two Danish screening programs was 2.6 saved breast cancer deaths per overdiagnosed case. Screening can affect only breast cancers diagnosed in screening age. Due to high breast cancer incidence after age 70, only one-third of breast cancer deaths after age 50 could potentially be affected by screening. Increasing upper age limit could be considered, but might affect benefit-to-harm ratio negatively.

## Introduction

In the late 1980s, randomized controlled trials showed that screening mammography could reduce breast cancer mortality, especially in women aged 50–69 years [1–3]. In Denmark, this led to initiation of two regional, population-based service screening programs in 1991 [4] and in 1993 [5]. Women aged 50–69 years were personally invited to screening free of charge every second year. The targeted women constituted 20% of Danish women in this age group.

In the following years, attempts to start organized screening elsewhere failed, and opportunistic screening was limited [6]. Nationwide service screening took off in 2008 [7], and rollout was completed in 2010 [8]. This means that over a period of 14–17 years, Denmark undertook a natural experiment, where 20% of women aged 50–69 years were invited regularly to screening while the remaining 80% were not.

In most settings, evaluation of service screening is difficult because there is no non-screened control group to compare with. In this context, the natural experiment puts Denmark in a special situation. Denmark furthermore has long-standing health registers. The combination of the natural experiment and register data allowed us to evaluate the impact of service screening. As an innovative approach in epidemiology, we used the difference-in-differences method, and undertook a number of studies on breast cancer mortality and overdiagnosis. In light of the continued debate about the value of breast cancer screening, we here summarize our findings. This allows for a comprehensive view of the impact of two independent, organized screening programs on both breast cancer mortality and overdiagnosis. Based on our findings, we discuss new aspects of the public health implications of breast cancer screening.

## Materials and methods

### Breast cancer screening

In Copenhagen, the program started in April 1991 [4]. Women aged 50–69 years were invited to a central clinic. At first screen women had two-view mammography. During the first 10 years of the program, women with fatty breast tissue had one-view mammography at subsequent screens, while women with mixed/dense breast tissue had two views. From the early 2000s, all women had two-view mammography. Mammograms were read independently by two radiologists. In the first four invitation rounds, 78% of invited women participated at least once [9].

In Funen, the program started in November 1993 [5]. Women currently aged 50–69 years were invited to a mobile unit/central clinic. Screening procedures were as in Copenhagen, and in the first four rounds 90% participated at least once [9].

### Difference-in-differences method

Materials and methods have been described in detail previously [10–13]. In short, in the difference-in-differences method, an outcome is compared between the exposed population and the population living in the same area prior to exposure, and controlled for change in outcome over the same time in the population living in another area where no exposure took place. To avoid selection bias, in our studies exposure was invitation to screening and not participation in screening.

**Fig. 1:**
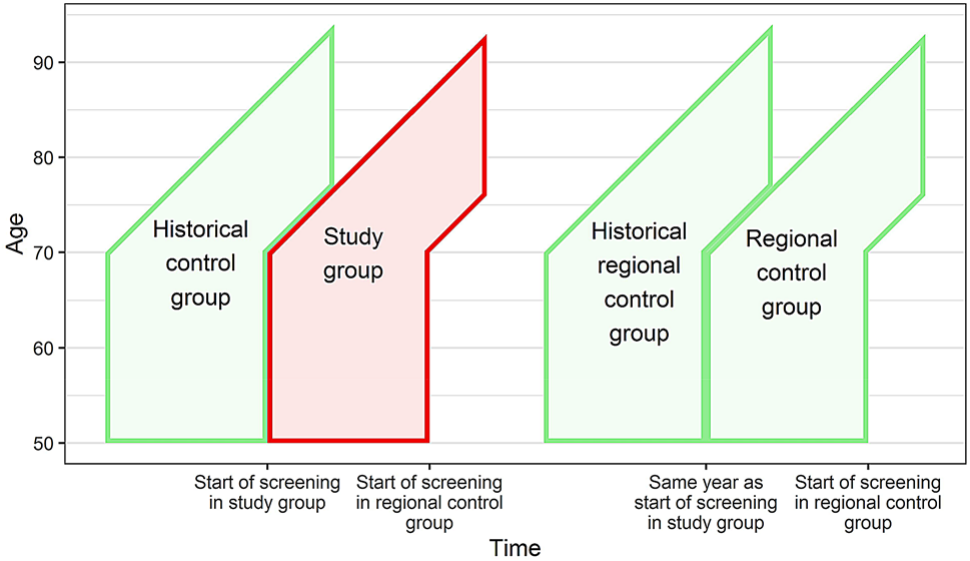
Design of Danish difference-in-differences cohort studies of impact of service breast cancer screening

For each analysis we formed four cohorts: study group including women in the target group for screening in the region offering screening; historical control group including women at similar age in the same region in the pre-screening period; regional control group including women at similar age in the rest of Denmark not offering screening; and regional–historical control group including women at similar age in the rest of Denmark in the pre-screening period [10]. To capture the full effect of screening, we followed older birth cohorts past the time where screening started in the younger birth cohorts in the regional control group, Fig. 1.

### Breast cancer mortality analysis

In the study group, women free of breast cancer on their first date of invitation to screening contributed person-years and breast cancer deaths from first date of invitation until death, emigration, or end of follow-up, whichever came first. The three control cohorts were constructed in similar ways, with a pseudo-date of first invitation allocated in accordance with invitations in the study group. This design is called the follow-up model [14].

However, for a longer follow-up period the cohorts will accumulate many new breast cancers after end of screening age. These breast cancers are, by definition, unaffected by screening, and deaths in these cases should be excluded from analysis. This design is called the evaluation model [14]. While it is straightforward to exclude these deaths, it is more difficult to define person-years at risk of death in post-screening age from breast cancers diagnosed in screening age. Traditionally, person-years for all women followed up are used in the evaluation model, but we consider it more appropriate after screening age to use only person-years in women diagnosed with breast cancer in screening age, because only these women can contribute to the numerator [12].

### Overdiagnosis analysis

Screening moves time of diagnosis forward, and this affects breast cancer incidence: a prevalence peak is expected during first screen; artificial aging due to diagnosis at earlier age in subsequent screens; a compensatory drop in first post-screen years, whereafter incidence is expected to return to the non-screening level [15, 16]. Overdiagnosed breast cancers are defined as cases that would in absence of screening not have become clinically manifest in the women’s life time [17], meaning that the excesses in incidence have not been compensated by the deficit. While there is consensus about this definition, various denominators have been used in quantification of overdiagnosis [18]. We focused on cumulative breast cancer incidence from first invitation up to 8 years or more after end of screening age, when the cohorts have experienced prevalence peak, artificial aging, and compensatory drop. As both invasive breast cancer and ductal carcinoma in situ (DCIS) can be diagnosed at screening, we included both [13].

In the study group, women free of breast cancer on first date of invitation to screening contributed person-years and incident breast cancers from first date of invitation until diagnosis of breast cancer, death, emigration, or end of follow-up, whichever came first. The three control groups were constructed similarly.

### Statistics

Breast cancer mortality or incidence rates were calculated for the study group and for the three control groups. The effect of screening was estimated as the ratio between the rate ratios ((study group/historical control group)/(regional control group/regional–historical control group)). The ratio of rate ratios (RRR) and the 95% confidence intervals (CI) were estimated with Poisson regression controlling for current age in 5-year age groups. In our overdiagnosis study, we included women born as far back as 1907. We therefore started out by testing for a possible interaction between region and period. As separating the interaction between period and region from the effect of screening is not possible for women of screening age, we used data from the respective birth cohorts from the 5-year age group below screening age for this purpose. Analyses were conducted using SAS, in latest studies version 9.4 (SAS Institute, Cary, NC).

## Results

### Breast cancer mortality

In our longest follow-up, the Copenhagen and Funen study groups together accumulated 1,551,465 person-years and 911 breast cancer deaths. During the first 10 years of the Copenhagen program, we saw a 25%, RRR 0.75 (95% CI 0.63–0.89), decrease in breast cancer mortality in the study group invited to screening compared with the change expected in the absence of invitation to screening [10], Table 1. A decline of 22%, RRR 0.78 (95% CI 0.68–0.89), was seen during the first 13 years of the Funen program [11]. The longer-term follow-up of the Copenhagen program included 16 years in the younger birth cohorts and 23 years in the older birth cohorts. A decrease in breast cancer mortality of 14%, RRR 0.86 (95% CI 0.77–0.97), was seen, when all women in post-screening age contributed person-years, and of 20%, RRR 0.80 (95% CI 0.71–0.90), when only screening-age breast cancer patients actually under risk of death in post-screening age contributed person-years [12].

**Table 1:** Breast cancer mortality 10–23 years after implementation of population-based screening in two regions of Denmark: estimates of screening effect

Person-years, breast cancer deaths, RRR (95% CI)	Follow-up model		Evaluation model	
	Copenhagen 1991–2001 [10]	Funen 1994–2007/08 [11]	Copenhagen 1991–2007/14 [12]	
			Person-years for all women	Person-years in post-screening age for breast cancer cases only
Study group				
Person-years	430,823	870,465	977,000	681,000
BC deaths	223	413	498	498
Regional control group				
Person-years	4,396,417	7,096,056	9,366,000	6,733,000
BC deaths	2333	4246	4848	4848
Historical control group				
Person-years	634,224	828,508	1,407,000	874,000
BC deaths	438	566	855	855
Regional–historical control group				
Person-years	4,055,004	6,151,011	7,031,000	4,809,000
BC deaths	2133	4111	3640	3640
RRR* (95% CI)	0.75 (0.63–0.89)	0.78 (0.68–0.89)	0.86 (0.77–0.97)	0.80 (0.71–0.90)

RRR, age-adjusted ratio of rate ratios; CI, 95% confidence intervals; BC, breast cancer

*Age adjusted

### Overdiagnosis

From the Copenhagen program, we included women aged 56–70 years at first invitation as they could be followed for at least 4 years in post-screening age [13]. In total, breast cancer, including DCIS, incidence was 6%, RRR 1.06 (95% CI 0.90–1.25) higher in the study group than expected based on incidence in the control groups, Table 2. Considerable variation was seen across the phases of screening: an excess incidence of 106%, RRR 2.06 (95% CI 1.64–2.59) during first screen; of 4%, RRR 1.04 (95% CI 0.85–1.27) during subsequent screens; and of a deficit of 20%, RRR 0.80 (95% CI 0.65–0.98) during the first 4 years of post-screening age. The birth cohorts of women aged 60–70 years at first invitation could be followed from first invitation to at least 8 years in post-screening age, where there was a 3.4%, RRR 1.034 (95% CI 0.86–1.25) increased incidence.

**Table 2:** Breast cancer incidence, including ductal carcinoma in situ (DCIS) by time since first invitation to population-based screening in two regions of Denmark: estimates of overdiagnosis

	Copenhagen [13]	Funen [13]
Age at entry	56–70 years	59–70 years
Study group		
Person-years	456,499	323,363
BC cases, incl. DCIS	2002	1277
Regional control group		
Person-years	4,173,549	2,768,352
BC cases, incl. DCIS	14,410	9898
Historical control group		
Person-years	909,875	359,426
BC cases, incl. DCIS	2639	1085
Regional–historical control group		
Person-years	3,999,172	2,731,457
BC cases, incl. DCIS	10,323	7635
RRR* (95% CI)		
Total	1.06 (0.90–1.25)	1.01 (0.93–1.10)
Prevalence screen	2.06 (1.64–2.59)	1.84 (1.46–2.32)
Incidence screens	1.04 (0.85–1.27)	1.14 (0.98–1.32)
0–3 years post-screening age	0.80 (0.65–0.98)	0.67 (0.55–0.81)
4–7 years	0.91 (0.75–1.16)	0.78 (0.64–0.96)
8+ years	0.99 (0.77–1.29)	0.98 (0.73–1.36)
Cumulative from first invitation to 8+ years post-screening age	1.034 (0.86–1.25)	1.007 (0.91–1.12)
Cumulative from first invitation to 8+ years post-screening age, pooled	1.023 (0.97–1.08)	

RRR, age-adjusted ratio of rate ratios; CI, 95% confidence intervals; BC, breast cancer; DCIS, ductal carcinoma in situ

*Age-adjusted

From the Funen program, only women aged 59–70 years at first invitation could be followed for at least 4 years in post-screening age. In total, breast cancer incidence in the study group was 1%, RRR 1.01 (95% CI 0.93–1.10), higher than expected from the control groups, with variation across phases of screening; 84%, RRR 1.84 (95% CI 0.46–2.32), during first screen; 14%, RRR 1.14 (95% CI 0.98–1.32), during subsequent screens; and a deficit of 33%, RRR 0.67 (95% CI 0.55–0.81), during the first 4 years in post-screening age. The birth cohorts of women aged 63–70 years at first invitation could be followed for at least 8 years in post-screening age, where there was a 0.7%, RRR 1.007 (95% CI 0.91–1.12), increased incidence. The pooled estimate from the two programs of cumulative breast cancer incidence from first invitation to at least 8 years after end of screening age was 2.3%, RRR 1.023 (95% CI 0.97–1.08).

## Discussion

### Main findings

Population-based personal invitation to biennial breast cancer screening in Denmark for women aged 50–69 years was associated with a long-term 20% reduction in mortality from breast cancers diagnosed in screening age, and an increased breast cancer incidence of 2.3%. These experiences derived from two screening programs where 78 to 90% of invited women were screened at least once.

### Other studies

Service breast cancer screening started based on randomized controlled trials when trial populations had been followed for about 10 years. In the meantime, trial data have become available for longer periods. The Swedish Two-County trial began in 1977–1978 and lasted 7 years. When followed up until 2005–2006, a 27% lower breast cancer mortality was seen in the active compared with the passive population, relative risk 0.73 (95% CI 0.59–0.89) [19]. The remaining Swedish trials started in Malmö in 1976–1978; Stockholm in 1981; and Göteborg in 1982. When data were followed until end of 2007, breast cancer mortality was 15% lower in the screening than in the control arm, relative risk 0.85 (95% CI 0.73–0.98) [20]. Our estimated long-term impact of service screening of a 20% reduction in breast cancer mortality is well in accordance with the long-term data from the Swedish trials showing reductions of 15 to 27%, respectively.

The Canadian trials started in 1980 targeting women aged 40–49 and 50–59 years, and screening centers closed in 1988. Initially, no impact was seen on breast cancer mortality, and a similar pattern was seen when the trial populations were followed until end of 2005 for deaths from invasive breast cancers diagnosed during the screening period, hazard ratio 1.05 (95% CI 0.85–1.30) [21]. One could speculate that in the absence of a short-term effect on breast cancers diagnosed during the screening period, no long-term effect on these cancers would be expected.

Most screening trials were not suitable for evaluation of overdiagnosis, as control groups were offered screening after trial completion. This was not the case in one Malmö trial, where the screening group was invited in 1976–1986, and both screening and control groups were followed until end of 2001. In women born 1908–1932, breast cancer incidence was 10% higher in the screening than in the control group, indicating a 10% overdiagnosis [22]. It should be noted though that the excess incidence derived mostly from women born in 1908–1912. They were screened up to an average age of 76.5 years, which left limited time for the compensatory drop to materialize as life expectancy was 80.8 years. A 1%, relative risk 1.01 (95% CI 0.85–1.19), overdiagnosis was seen in women born 1918–1922 and screened up to 70 years [23]. Our finding of a 2.3% overdiagnosis is compatible with these Swedish data.

In Canada, screening was not offered to control groups at trial completion. At long-term follow-up, there were 106 more breast cancers in the screening than in the control arm. As 106 cases were equal to 22% of screen-detected invasive cancers, the authors concluded that 22% of screen-detected cancers were overdiagnosed [21]. However, shortly after the Canadian trials stopped, service screening was implemented for women aged 50–69 in the majority of Canadian provinces from which the trial populations were recruited. This would not leave time for the compensatory drop to materialize in the screening arm of the trial population [23]. Combining our estimate of overdiagnosis of 2.3% [13] with the distribution of breast cancers in screening age into screen-detected, interval cancers, and cancers in non-screened women [24], overdiagnosed cases in Copenhagen and Funen constituted roughly 6–7% of screen-detected breast cancers including DCIS.

The Danish breast cancer mortality data have been analyzed also by other researchers investigating average annual change in crude age-specific breast cancer mortality from 1982 to 1991 before screening started, and from 1997 to 2006 some years after screening started [25]. From both periods, the annual changes were similar between screening and non-screening regions, and the authors “were unable to detect any effect of the Danish screening programs on breast cancer mortality.” This conclusion is problematic for two reasons. First, deaths in breast cancers diagnosed before screening were included in the analysis. We estimated these deaths to constitute 39% of deaths. Second, average annual change is the slope of the line, but the researchers ignored a decrease in level of the screening regions’ line in the time period 1991–1996, which they excluded from their analysis. We estimated the decrease in this time period to be 13%, RRR 0.87 (95% CI 0.79–0.95), as compared with the line in non-screening regions. Given that the 13% is based on data contaminated by deaths in cases diagnosed prior to screening, it is compatible with our findings.

Overdiagnosis has been estimated also by other researchers based on the Danish data [26]. First, overdiagnosis was estimated from absolute size of the difference in changes in breast cancer incidence between screening and non-screening regions. Second, it was assumed that screening affected non-advanced cancer only, a calculation somewhat similar to the first approach was undertaken, and an estimate for DCIS was added. This last calculation resulted in an overdiagnosis of 48.3%, leading authors to conclude that “1 in every 3 women aged 50–69 years diagnosed with breast cancer was overdiagnosed in the screening area.” These calculations were affected by several methodological mistakes [27, 28].

No other country or region undertook a natural experiment as the Danish one. The most similar settings were the public health trial in Finland, where to start with only every second birth cohort was invited and which showed a deficit of 24%, relative risk 0.76 (95% CI 0.53–1.09), in breast cancer mortality in early compared with later invited cohorts [29], and the municipality-based implementation of screening in the Netherlands, where breast cancer mortality rate 20 years after introduction of screening was reduced by 30% for women aged 55–74 years [30]. Several other studies have been undertaken of the outcome of service screening; 36 cohort studies and 17 case–control studies were identified in a recent review of European studies. It is noteworthy though that the authors of this review concluded that while the data provided “evidence that organised screening reduces breast cancer mortality in all European regions … [a] wide range of estimates indicates large differences in the evaluation designs between studies, rather than in the effectiveness of screening” [31].

### Strengths and limitations

It is a strength of the difference-in-differences method that it allows for control of both region and time. It is a limitation that the method does not directly allow for control of a possible interaction between region and time, i.e., if breast cancer treatment improved more rapidly over time in study region than in control region. This has been hypothesized, e.g., for Norway, where multidisciplinary teams were implemented simultaneously with screening [32]. In Denmark, interaction is unlikely to explain the decrease in breast cancer mortality associated with invitation to screening. First, diagnostic and therapeutic strategies for breast cancer patients have been organized nationwide since 1977 with uniform guidelines for histopathology, surgery, radiotherapy, and systemic therapy [33]. Second, Funen started multidisciplinary teams in 1979 [34], long before screening, and long before multidisciplinary teams were introduced in the rest of Denmark. Third, results were fairly similar from the capital Copenhagen and from the mixed rural–urban area of Funen. Finally, no interaction was found between region and time in the pre-screening period 1970–1989 [35]. It is on this basis reasonably to assume a causal association between invitation to screening with the high coverage seen in the Danish programs and the observed decrease in breast cancer mortality. In the overdiagnosis study, we included birth cohorts back to women born in 1907, and breast cancer incidence data from below screening age were used to control for interaction between region and time.

As only women diagnosed with breast cancer in screening age can contribute to the numerator in the evaluation model, we consider person-years accumulated by these patients to be the most appropriate denominator, but we cannot exclude that these person-years are to some extent affected by lead time. From Copenhagen, the short-term effect of screening on breast cancer mortality estimated in the follow-up model was RRR 0.75 (95% CI 0.63–0.89), while the long-term effect estimated in the evaluation model was RRR 0.80 (95% CI 0.71–0.90). One might have expected the opposite pattern. This might indicate that the short-term effect of screening was stronger than the long-term effect, and it might reflect that lead time could have affected the estimates differently. It should though be taken into account that the two estimates had largely overlapping confidence intervals.

Follow-up time was a limitation in our overdiagnosis study where only birth cohorts first invited to screening at age 60 years and above had observations up to at least 8 years in post-screening age. This could indicate an underestimation of overdiagnosis, as possible cases overdiagnosed in the age span 50–59 years were not included. It should though be taken into account that only screen-detected cases with a lead time of over 10 years would have been unaccounted for in our analysis.

### Public health implications

In high-income countries, breast cancer mortality has decreased over the past 20–20 years. In Denmark, it peaked in the late 1980s with an age-standardized rate of 50 per 100,000 (Nordic standard population), and it decreased steadily thereafter to 32 per 100,000 in 2016 [36]. This trend in the age-standardized breast cancer mortality has been fairly equal in countries starting service screening early, as in Sweden, or late, as in Norway [37]. Nevertheless, our studies demonstrated that in well-organized programs, service screening achieves about the same reduction in breast cancer mortality as seen in the randomized controlled trials. So, why do we have these seemingly contradictory observations? Our study of the service screening program in Copenhagen [12] provided an answer to this question. The effect of screening is limited to patients diagnosed in screening age, and in the Copenhagen cohort we found that only 37% of breast cancer deaths expected in absence of screening, derived from patients diagnosed in the screening age 50–69 years [12]. The majority of breast cancer deaths counted in the overall age-standardized rates therefore occur in women diagnosed when they are already above the upper screening age of 70 years.

So, should the upper age limit for screening be increased beyond 70 years to further reduce breast cancer mortality? Data from the randomized controlled trials demonstrated a decrease in breast cancer mortality from screening in the age group 70–74 years [3], and in Europe, screening up to 75 years is currently recommended in, e.g., the Netherlands [38] and Sweden [39]. While there are no data from randomized controlled trial for women above the age of 75 years, a recent observational study of 70-year-old, recently screened, US women enrolled in Medicare with a comorbidity score < 1 found no benefit of screening beyond the age of 75 years [40].

The impact of screening on breast cancer mortality may thus be enhanced by increasing the upper age limit to 75 years. However, a potential benefit on breast cancer mortality has to be balanced against a potential harm of overdiagnosis. The benefit-to-harm ratio is defined as the number of breast cancer deaths saved divided by the number of overdiagnosed cases [41]. Based on the reported studies, the Danish benefit-to-harm ratio was 2.6 [42]. The benefit-to-harm ratio might still be favorable in Denmark if the upper screening age was extended to 75 years, but as the remaining life expectancy at this age is 12.37 years [43], it would become a more delicate balance especially for women in areas and social groups with a low life expectancy. In light of the limited overdiagnosis of 2.3% found in the two early screening programs, it is noteworthy that during the nationwide rollout of service screening to all Danish women aged 50–69 years in 2008–2010, no increase the breast cancer incidence was seen beyond the prevalence peak [36].

Personalized screening targeted to women according to their individual breast cancer risk factors as previous benign breast lesions and reproductive history is suggested as a way to overcome the limitations of “one-size-fits-all” screening. In considering personalized screening, it should, however, be taken into account that the screening test itself is probably the best available tool to discriminate between high and low risk women. In the present nationwide Danish screening program with its “one-size-fits-all” biennial screening of women aged 50–69 years, the 2-year risk of breast cancer was 80 times higher in screen-positive than in screen-negative women [44]. In comparison, the ability of personalized screening models to discriminate between low and high risk women is 0.51–0.80, which is between none and moderate [45]. Another approach to personalized screening might be to target women based on the outcome of screening. In the two early Danish programs, the reduction in breast cancer mortality was 4 times larger in women not previously hospitalized than in women previously hospitalized with chronic diseases [46].

Personalized screening is an option under investigation. Firm evidence is needed in order to offer subgroups of women less or more screening than currently offered in the “one-size-fits-all” program. It is noteworthy that both in the American [47] and the European [48] trials on personalized screening, all women in the intervention groups, except women with the very lowest risk in Europe, are offered more intensive screening than presently offered to all women in Denmark.

## Conclusion

In breast cancer screening, Denmark undertook a natural experiment with two regions starting screening 14–17 years before the rest of the country. Combined with individual health records, this allowed for a thorough evaluation of service screening. A long-term 20%, RRR 0.80 (95% CI 0.71–0.90), reduction in breast cancer mortality was found, and an overdiagnosis of 2.3%, RRR 1.023 (95% CI 0.97–1.08). The main limitation of service screening is that it can affect only women diagnosed with breast cancers in the age-window targeted for screening, and breast cancer deaths in these women constitute only about one-third of all breast cancer deaths.

## Abbreviations

CI: Confidence intervals
DCIS: Ductal carcinoma in situ
RRR: Ratio of rate ratios
